# Flooding Irrigation Weakens the Molecular Ecological Network Complexity of Soil Microbes during the Process of Dryland-to-Paddy Conversion

**DOI:** 10.3390/ijerph17020561

**Published:** 2020-01-15

**Authors:** Xiaoxiao Li, Qi Zhang, Jing Ma, Yongjun Yang, Yifei Wang, Chen Fu

**Affiliations:** 1 School of Environment Science and Spatial Informatics, China University of Mining and Technology, Xuzhou 221008, Jiangsu, China; lixiaoxiao@cumt.edu.cn (X.L.); zhangqi2019@cumt.edu.cn (Q.Z.); y.yang@cumt.edu.cn (Y.Y.); yifeiwang@cumt.com (Y.W.); 2 Low Carbon Energy Institute, China University of Mining and Technology, Xuzhou 221008, Jiangsu, China; jingma2013@cumt.edu.cn

**Keywords:** land consolidation, soil microorganism, high-throughput sequencing, molecular ecological network, bacterial diversity, land use conversion

## Abstract

Irrigation has been applied on a large scale for the improvement of grain yield per hectare and production stability. However, the dryland-to-paddy conversion affects the ecological environment of areas of long-term dry farming, especially soil microorganisms. Little attention has been paid to the changes in microbial communities and the interactions between their populations in this process. Therefore, in this paper, the compositions and diversity of soil bacterial and fungal communities were explored through a combination of high-throughput sequencing technology and molecular ecological network methods using bacterial 16S rRNA and fungal ITS. The results showed that: (1) both the abundance and diversity of soil bacteria and fungi decreased in a short time, and the abundance of *Actinobacteria*, *Firmicutes* and *Olpidiomycota* varied greatly. (2) Compared to dry land, the modular structure of interaction networks and interspecific relationships of bacterial and fungal communities in paddy soil were simpler, and the network became more unstable. A cooperative relationship dominated in the molecular ecological network of bacteria, while a competitive relationship was dominant in the network of fungi. *Actinobacteria* and *Firmicutes* were the dominant bacterial species in dry land and paddy field, respectively. *Ascomycota* was dominant in the fungal communities of both dry land and paddy field. (3) The change in soil environmental factors, such as pH, electrical conductivity (EC), organic matter (OM) and available potassium (AK), directly affected the soil microbial community structure, showing a significant correlation (*p* < 0.05). These environmental factors also influenced the dominant microbial species. Microorganisms are the most important link in the carbon and nitrogen cycles of soil, and a large-scale dryland-to-paddy conversion may reduce the ecological stability of regional soil.

## 1. Introduction

Land use conversion is an important factor causing global environmental changes [1,2,3,4]. Most importantly, the use and management of farmland influences the carbon and nitrogen cycles of terrestrial ecosystems [5]. The varied utilization mode of farmland has affected the carbon storage and carbon flux of a regional ecosystem, and the carbon emission caused by this change shows an important impact on global warming [6,7,8,9,10]. Driven by grain demand and economic benefits, a large area of rain-fed farmland in semi-humid regions has been converted into irrigated agriculture [11,12]. This conversion not only changes the agricultural farming pattern, but also changes the natural cover and properties of underlying surfaces, resulting in changes in soil properties and ecological processes [8,13]. Furthermore, large-scale living conditions including regional microclimate and surface runoff will be affected [14]. The large-scale expansion of irrigation agriculture threatens the security of groundwater resources, intensifies the evaporation from the land surface, aggravates extreme high-temperature climates in summer, and accelerates changes in global climates [11,15]. For a farmland ecosystem, the conversion from dryland to paddy field destroys the stability of long-term dry-farming soil, inevitably and subtly affecting the regional ecological environment, especially the carbon effect [14].

Microorganisms are the most active component of soil ecosystems [16,17,18,19], which participate in geochemical cycles being the main decomposers and maintainers [20]. Dry land and paddy field are distinct land use patterns, and paddy field is the main source of agricultural greenhouse gases [21,22], threatening the global environment. Land use conversion has a significant impact on the soil properties and environment [7], including the evolution of soil microbial community structure and the carbon and nitrogen cycles of the ecosystem [23]. Large-scale dryland-to-paddy conversion changes the regional soil environment [24] and affects the physicochemical characters and biological processes of the soil, thus leading to great changes in the carbon cycle of farmland. Soil microorganisms are the engines of the carbon and nitrogen cycles [25,26,27]. Soil microbial community structure controls the carbon and nitrogen transformation in an ecosystem through participating in the decomposition of organic matter and formation of humus [27,28,29,30]. Soil microorganisms can rapidly respond to environmental changes, while microbial structure and abundance can be used as the important indicator to assess the soil quality [30]. The interactions between soil microorganisms and the collinear relationships between microorganisms and the environment can be clarified by molecular ecological network analysis [31,32,33,34], which provides a new way to reveal the stability and complexity of ecological processes and ecosystem functions [35,36,37,38,39]. Zhou et al. reported that in an environment with an elevated CO_2_ level, the molecular ecological networks of soil microorganisms changed to a great extent, and the interactions between different populations were significantly changed [40]. Liu et al. analyzed the molecular ecological network structure of microbial fungi in potato farmland soil, and found that the network structures, topological properties and key species of low-yield and high-yield soils were significantly different [41]. However, the changes in soil microbial molecular ecological network during the land use conversion process from dryland to paddy field, as one of the most important ecological processes, have never been documented.

Although soil environment is always a concern in agricultural production, the microscopic environmental effects of dryland-to-paddy conversion, especially the coupling mechanism between soil microbial communities and the environment, are still being unraveled. The changes in soil microbial community structure and the interactions between various species during the process of drought-to-water conversion urgently need to be studied. In this study, high-throughput sequencing technology and ecological network analysis methods were used to determine the properties of bacterial 16S rRNA and fungal ITS, to monitor the changes in farmland soil environment after dryland-to-paddy conversion for the exploration of evolution of soil microbial community structure. Furthermore, the changes in interaction networks between soil bacterial and fungal communities and their mechanism were studied, and the differences and similarities between bacterial and fungal network structure before and after the process of dryland-to-paddy conversion were investigated. This work focuses on improving the regional ecological environment and future agriculture, and on providing new strategies for coping with global climatic changes.

## 2. Materials and Methods

### 2.1. Study Area

The experimental region is located on the Nanhu Campus of China Mining University (117.2N, 34.2E), Xuzhou City, Jiangsu Province. This region exhibit a warm semi-humid monsoon climate, with an annual temperature of 14 °C, annual frost-free period of 209 days, and annual precipitation of 833 mm [42]. It is hot and rainy in the same season, and wheat and corn are grown on alternate years. This region is located in the Huang-Huai-Hai Plain, and is relatively flat. The groundwater level in this area was high, and the problem of soil salinization is serious. Because the natural conditions meet the needs for rice cultivation, in the last decade, large-scale land consolidation is carried out for the alternate growing of wheat and rice for higher and more stable yields of grains.

This study was set up in pots in the fields of the Land Science Research Center of Nanhu Campus of China University of Mining and Technology on June 13, 2018. Wheat and corn had been planted in the soil for the previous five consecutive years. The soil type was cinnamon soil and light loam in texture, with soil particle composition of 15.12 (<0.001 mm): 22.34 (0.001–0.005 mm): 9.15 (0.005–0.01 mm): 53.39 (>0.01 mm) [43]. When the soil was acquired, the original cultivated layer had not been destroyed. We set up two groups of potted experiments, and each group had 10 parallel experiments. In one group, Nonghua-101 maize was cultivated in dry land (DL), and five maize plants, with row spacing of 300 mm and plant spacing of 200 mm, were planted in each pot. In the other group, Wuyugeng-31 paddy rice was cultivated in paddy field (PF). Since Xuzhou belongs to the semi-humid region, the dry farming in this area is mainly rain-fed agriculture, and agricultural production based solely on natural precipitation as the source of water. In this experiment, the DL was the same as the local dry farming habit and was not irrigated. The Wuyugeng-31 seeds were previously soaked in water for 5 days, and four seedlings, with row spacing of 300 mm and plant spacing of 200 mm, were planted in each pot. Afterwards, the paddy irrigation maintains average water-layer depth of no less than 40 mm. The irrigation water was tap water, in accordance with Chinese agricultural irrigation standards. All the containers were polyethylene pots with a height of 60 cm, diameter of 40 cm, and soil-layer thickness of 40 cm.

### 2.2. Soil Sample Collection and Analysis

After 146 days of potted cultivation, soil samples in the DL and PF cultivation layers at 0–20 cm were collected with a five-point sampling method on November 6, 2018. Ten soil samples were respectively collected from parallel pot experiments in DL and PF. The total weight of each sample was about 1000 g. The soil samples were immediately packed and sealed in sterile polyethylene bags, and frozen at −20 °C for further treatment. A half of the fresh soil samples were directly tested for biodiversity analysis. The other samples were naturally dried. After gravel and animal and plant residues were removed, these samples were ground and sieved with a 2-mm sieve. The basic physical and chemical properties of the fine powders were measured.

The pH, electrical conductivity (EC), organic matter (OM), ammonium nitrogen (AN), nitrate nitrogen (NN), available potassium (AK), and available phosphorus (AP) of the soil samples were measured. In detail, pH and EC were measured by potentiometry (water:soil = 1:2.5). OM was measured with a potassium dichromate oxidation-outer heating method. AN was measured by ultraviolet spectrophotometry after extraction with potassium chloride. NN was measured by ultraviolet spectrophotometry after extraction with calcium chloride. AP was measured by molybdenum-antimony-scandium colorimetry after extraction with ammonium bicarbonate. AK was measured by flame photometry after extraction with ammonium acetate (FP640, Jingke, Shanghai, China) [24,43].

### 2.3. DNA Extraction, PCR Amplification, and Illumina Miseq Sequencing

According to the instruction of FastDNA™ SPIN kit (MP Biomedicals, Solon, OH USA), DNA of 20 farmland soil samples was extracted. Afterwards, the V4 and V5 regions of bacterial 16S rDNA were amplified using the primers 515F (5′-GTGCCAGCMGCCGCGGTAA-3′), and 907R (5′-CCGTCAATTCMTTTRAGTTT-3′). PCR amplification from soil samples of dry land and paddy field was conducted with the standard fungus ITS1 primers ITS5F (5′-GGAAGTAAGTCGTAACAAGG-3′) and ITS1R (5′-GCTGCGTTCTTCTCGATGC-3′). PCR amplification was as follows: 1 cycle of pre-denaturation at 98 °C (2 min), then 25 cycles of; denaturation at 98 °C (15 s), annealing at 55 °C (for 30 s), extension at 72 °C (30 s), then 1 cycle of extension at 72 °C (5 min), and cooling to 10 °C. The products of PCR amplification were separated by 2% agarose gel electrophoresis. The target sequences were recovered with a gel extraction kit (Axygen, San Francisco, CA, USA). Following the preliminary electrophoresis quantitation results, the sequences recovered were quantified using a microplate reader (FLx800, BioTek, Winooski, VT, USA) apparatus with Quant-iT PicoGreen dsDNA Assay Kit fluorescent reagent. The samples were then mixed in equal proportion. The sequencing library of soil bacteria and fungi were prepared with the TruSeq Nano DNA LT Library Prep Kit (Illumina, San Diego, CA, USA). The constructed library was quantified by Qubit and Q-PCR, and then was sequenced with HiSeq2500 PE2500 (Illumina) [43,44].

### 2.4. Analysis of Soil Microbial Molecular Ecological Network

Based on the theory of random matrix, the molecular ecological networks of soil microorganisms in dry land and paddy field were constructed separately. The construction and parameters setting were completed on the Mena platform (http://ieg4.rccc.ou.edu/mena). After the data were acquired through high-throughput sequencing, the logarithms of operational taxanomic unit (OTU) data (log10) were transformed into a correlation matrix. Based on the Pearson correlation coefficients, the matrix was transformed into a similarity matrix. Using random matrix theory (RMT), with appropriate threshold values, an adjacency matrix was generated from the similarity matrix. The connection strength between each pair of nodes was coded with the adjacency matrix. The ecological community was predicted by analyzing the nearest distance distribution of the eigenvalues of the correlation matrix [35]. The network was visualized by Cytoscape. Each ecological network was divided into several modules, and each module was a functional unit of the microbial system. In the structural diagram of the network, the nodes represented the populations in the microbial community, and the connecting lines between nodes represented the interactions between populations. Based on the modules in the network, the influence of soil environmental factors on the ecological network was considered. The correlations between microbial interaction networks and environmental factors were analyzed by Pearson correlation analysis methodology. Finally, Cytoscape 3.7.1 software was used to visualize the soil microbial molecular ecological networks in dry land and paddy field [24,45].

### 2.5. Data Statistic and Analysis

The Alpha diversity index was calculated based on the galaxy platform (http//:mem.rcees.ac.cn.8080/). The analysis of variance (ANOVA) and Pearson correlation analysis of soil physical and chemical properties before and after the conversion from dry land to paddy field were carried out with the SPSS19.0 software (IBM, Armonk, NY, USA). Based on the high-throughput sequencing results of bacteria and fungi, the molecular ecological network was constructed and characteristic parameters were calculated on the Mena platform (http://ieg4.rccc.ou.edu/mena). The network was plotted with the Cytoscape software. The topological structural diagram of the network was plotted with the Origin 9.0 software (OriginLab, Northampton, MA, USA). The correlations between soil physical and chemical factors and the network were analyzed with the ecodist package in R-Studio v7.2 (R Development Core Team, Vienna, Austria) in Mantel test software.

## 3. Results

### 3.1. Effects of Dryland-to-Paddy Conversion on Soil Microbial Community Structure

#### 3.1.1. Impact on Soil Microbial Community Diversity

**Figure 1:**
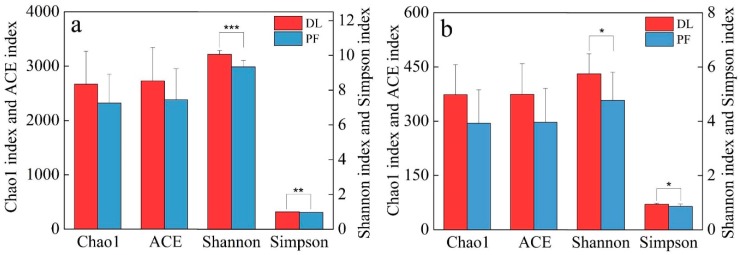
Alpha diversity indices of soil (**a**) bacteria and (**b**) fungi in different land use types. Note: DL is the soil sample of dry land before corn harvest, and PF is the soil sample of paddy field before rice harvest. The same for below.

The Alpha diversity results before and after dryland-to-paddy conversion are shown in Figure 1. In the short term after dryland-to-paddy conversion, the soil microbial diversity was significantly reduced, and the Simpson, Chao1, ACE and Shannon indexes of paddy field were lower than those of dry land. Compared to dry land, the bacterial diversity index of paddy soil was much lower, and the Shannon index was decreased by 0.62% to 11.99%. The average decline in the Shannon index for fungi was 17.04%. The dryland-to-paddy conversion largely affected the Shannon and Simpson indices. In the short term after dryland-to-paddy conversion, the soil microbial diversity was significantly reduced (*p* < 0.05).

**Figure 2:**
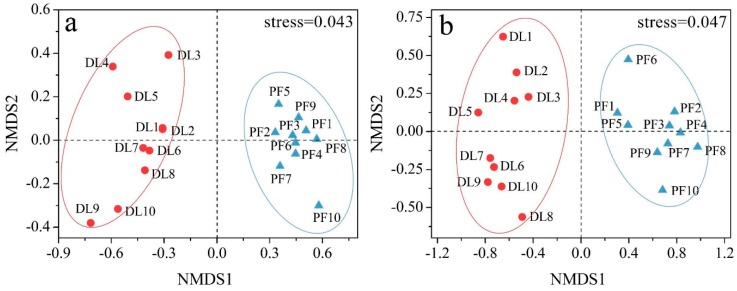
NMDS analysis results of soil (**a**) bacteria and (**b**) fungi at the genus level in different land use types.

Using the OTU classification, non-metric multidimensional scale (NMDS) was performed for β diversity analysis. The stress coefficient was lower than 0.05, indicating that the results were representative. The colored dots corresponded to different samples (or groups). The closer together two dots were, the higher the similarity between the microbial community structure of the two samples, and the smaller the differences. NMDS analysis results showed that the microbial communities of dry land and paddy field were greatly different, and the composition and structure of soil bacteria within the groups of paddy field were very similar (Figure 2).

#### 3.1.2. Impact on the Soil Microbial Community Composition

**Figure 3:**
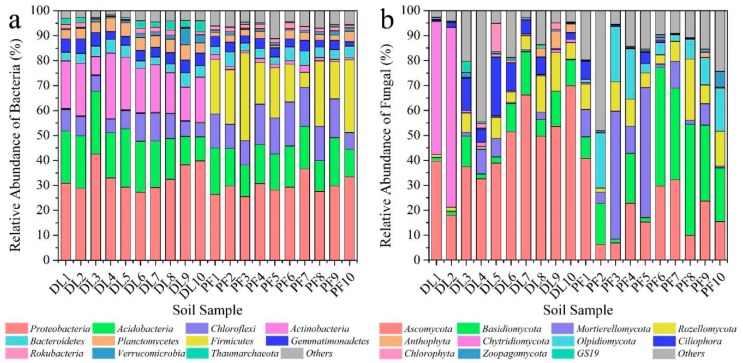
Abundance of soil (**a**) bacteria and (**b**) fungi communities for different land use types at the phylum level.

95% of the sequences of tested soil samples can be clearly identified and divided into 38 phyla of bacteria, of which Proteobacteria were the most abundant, accounting for about 31.5% (Figure 3a). Compared to dry land with loose soil, the paddy field possessed a significant anaerobic environment, resulting in the decline in abundance of *Acidobacteria*, *Actinobacteria*, *Gemmatimonadetes*, *Planctomycetes*, and other aerobic bacteria after the conversion from dry land to paddy field. Because *Actinobacteria* usually grows in neutral or alkalescent aerobic soil, out of the above listed bacteria [46], the abundance of *Actinobacteria* was significantly decreased from 17.94% to 2.97%, which is a 6.04 times difference. Under the flooded anaerobic environment after the conversion, the relative abundance *Chloroflexi* and *Firmicutes* in PF increased. Most notably, the abundance of *Firmicutes* was increased greatly, from 0.23% to 18.85%.

At the phylum level, 14 and 16 fungi were identified in dry land and paddy field, respectively. *Ascomycota* was the most abundant species, accounting for approximately 33.01% (Figure 3b). After the conversion from dry land to paddy field, the abundance of aerobic fungi such as *Ascomycota* and *Chytridiomycota* declined. For instance, *Chytridiomycota* was the dominant fungus in dry land, with an abundance of 13.26%, however its abundance declined to 0.1% in paddy field. In contrast, the abundance of *Basidiomycota, Mortierellomycota*, and *Olpidiomycota* increased. For example, the relative abundance of *Olpidiomycota* increased from 0.04% to 11.29%.

### 3.2. Effects of Dryland-to-Paddy Conversion on the Structure of Soil Microbial Molecular Ecological Networks

#### 3.2.1. Changes in OTU Topological Structures of Molecular Ecological Networks Generated During the Process of Dryland-to-Paddy Conversion

Based on RMT, the OTU values ≥ 150 for the ten bacterial samples and OTU values ≥ 44 for the ten fungal samples were selected as the input parameters, and the comparable selection thresholds of bacteria and fungi were set at 0.88 and 0.86, respectively, to generate molecular ecological networks. The specific topological parameters are shown in Table 1. The average connectivity, average path distance, average aggregation coefficient and modularity of all the constructed networks were greater than those of random networks, and the R^2^ values were > 0.75, indicating that the results were reliable [38]. In the bacterial and fungal networks, the numbers of nodes and connecting lines for dry land were greater than those of paddy field, indicating that the molecular ecological network became simpler after dryland-to-paddy conversion.

Moreover, the average degree, average path distance and connectivity of dry land were higher than those of paddy field, indicating that the relative connectivity of dry land molecular ecological network was more complex than that of paddy field, and the network nodes formed were closer. However, the module indices of bacterial and fungal networks were greater than those of dry land, indicating that short-term conversion from dry land to paddy field improved the resistance of microbial communities to external changes. For the bacterial network, the average clustering coefficient of dry land was slightly smaller than that of paddy field, indicating that the connectivity between each node of paddy field was enhanced. The average clustering coefficient of the fungal network was increased and the density was decreased after dryland-to-paddy conversion, which indicated that the connectivity between each node of paddy field was weakened, and the network was simpler than that of dry land. This may be related to the changes in soil environment or the reconstruction of microbial functional groups.

**Figure 4:**
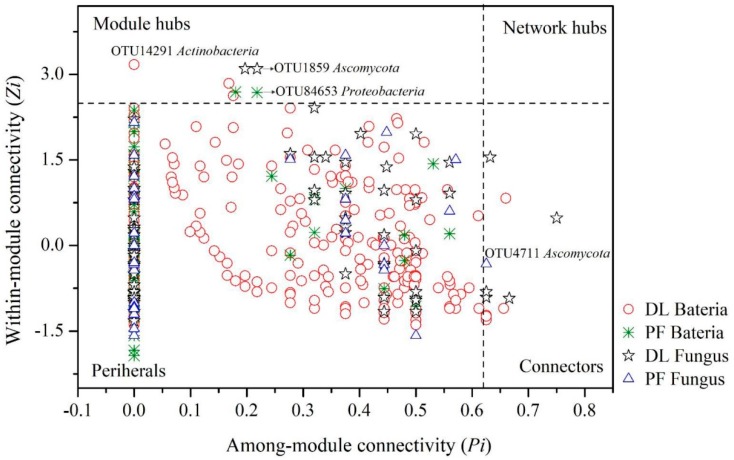
Z-P plots of molecular ecological networks for different land use types.

The connectivity within or between modules of molecular ecological networks can reflect the roles played by different OTUs. Generally, nodes with Zi values ≥ 2.5 or Pi values ≥ 0.62 are considered as critical species. The dry land bacterial ecological network consisted of four connecting nodes and three module hubs, while the paddy field bacterial ecological network consisted of only two module hubs. The dry land fungal ecological network consisted of four connection nodes and two module hubs, while the paddy field fungal ecological network consisted of only one connection node. Figure 4 shows that for the critical species in the module hubs and connectors, the nodes with the highest connectivity are located either above and or to the right side of the dashed line. For bacterial network, the critical species with the highest connectivity in dry land (*Actinobacteria*) and paddy field (*Proteobacteria*) were different. On the other hand, for the fungal network, the critical species with the highest connectivity in both dry land and paddy field is *Ascomycota*.

#### 3.2.2. Construction and Evaluation of Molecular Ecological Networks Generated during the Process of Dryland-to-Paddy Conversion

**Figure 5:**
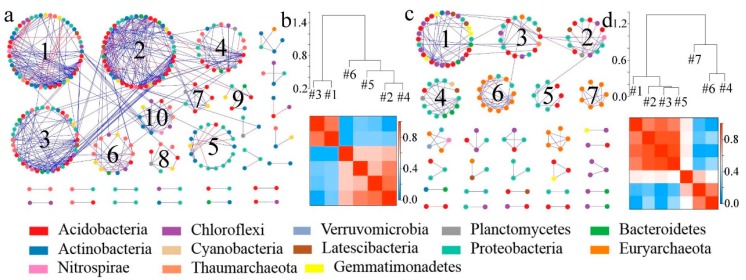
Soil bacterial molecular ecological networks and modular eigengene hierarchy structure in DL and PF. (**a**) Soil bacterial molecular ecological network in the DL; (**b**) soil bacterial modular eigengene hierarchy structure in the DL; (**c**) soil bacterial molecular ecological network in the PF; (**d**) soil bacterial modular eigengene hierarchy structure in the PF. Note: The connection lines represent the interactions between communities on the phylum level. The blue and red colors indicate positive and negative, respectively (the use of colors remains the same in the following sections).

**Figure 6:**
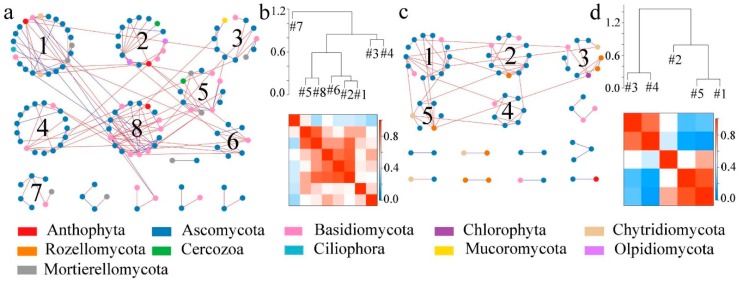
Soil fungal molecular ecological networks and modular eigengene hierarchy structure in DL and PF. (**a**) Soil fungal molecular ecological network in the DL; (**b**) soil fungal modular eigengene hierarchy structure in the DL; (**c**) soil fungal molecular ecological network in the PF; (**d**) soil fungal modular eigengene hierarchy structure in the PF.

Based on the results of soil microbial sequencing, four visualized molecular ecological networks (Figure 5a and Figure 6a) were constructed to illustrate the relationships between bacterial and fungal species in the soil after the conversion from dry land to paddy field. For the molecular ecological network of soil bacteria, there were 10 modules formed in the dry land network, and seven modules formed in paddy field. After the conversion from dry land to paddy field, there were less modules formed in the paddy field network than were formed in the dry land network (Figure 5b,d), and the connectivity between modules was reduced. After the conversion, soil bacteria tended to be more cooperative (blue), and the relationships between bacteria were looser compared to dry land (Figure 5a,c). Soil bacteria changed to adapt to the changes in soil environment caused by the conversion.

For the fungal molecular ecological network, a comparison of Figure 6b,d shows that the number of modules in the soil fungal molecular ecological network were reduced and the network became simpler after the conversion from dry land to paddy field. In Figure 6a, the connecting lines between nodes in the dry land network are significantly higher than those in the paddy field network (Figure 6c), and the nodes tended to be more competitive between each other (red), but the interactions between bacterial communities were significantly weakened. After the conversion, the relationships within and between modules became looser. *Ascomycota* dominated in the fungal networks of dry land and paddy field. The fungal network of dry land is more complex than that of paddy field. The disturbance generated by external environmental factors could not affect the whole fungal network in a short time, and the community structure was more stable. However, there are fewer nodes and connecting lines in the paddy field network and the stability was poor, which may be related to the stress on the soil after flooding.

### 3.3. Mechanism of Interactions Between Soil Microbial Community and Soil Environmental Factors

#### 3.3.1. Relationships between OTU Levels of Soil Microorganisms and Environmental Factors

For the screening of the dominant environmental factors affecting soil microbial community structure, OTU levels were selected for the Mantel test analysis on the soil microorganisms and their corresponding physical and chemical properties in dry land and paddy field. The results are shown in Table 2. Microorganisms were closely correlated with pH (*p* < 0.05). As an important index to the acidity, alkalinity and salinization degrees of soil, pH had a significant impact on the structural differentiation of bacteria and fungi. In addition, EC is another important factor affecting the microbial structure, and the bacteria and fungi had significant relationships with EC. Bacterial OTU had a significant positive correlation with OM, and paddy field fungus OTU had a significant positive correlation with AK. These indicated that the soil nutritional indices could affect the microbial community structure.

**Figure 7:**
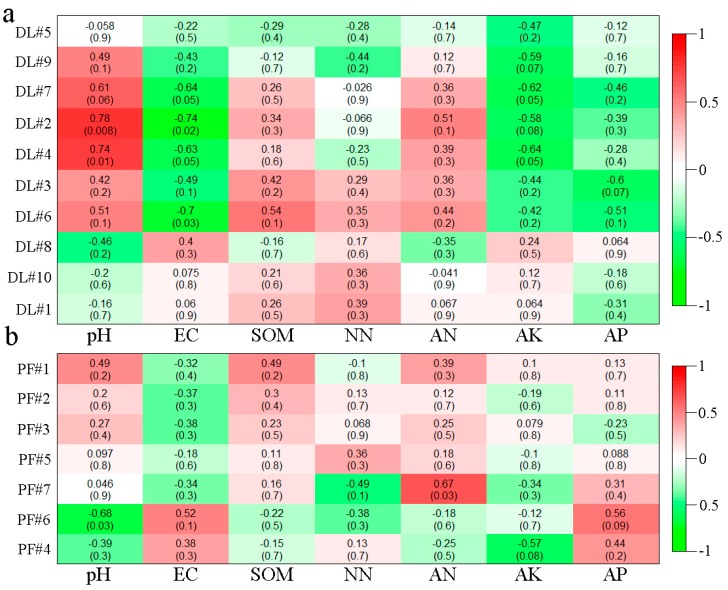
Heatmap analysis of bacteria module correlations with environmental factors in DL (**a**) and PF (**b**).

At the OTU level, the relationships between the modules with more than 5 nodes and the environmental factors in the bacterial molecular ecological network were analyzed by Heatmap. The as-derived Heatmap is shown in Figure 7. The results showed that the clustering OTU values of modules 2, 4 and 7 of dry land bacteria were positively correlated with pH, while they were negatively correlated with soil EC and AK (Figure 7a). On the other hand, among the seven modules formed by paddy field bacteria, module 6 was significantly negatively correlated with pH, module 7 was significantly positively correlated with AN, module 4 was significantly negatively correlated with AK, and module 6 was significantly positively correlated with AP (Figure 7b). The results showed that after the conversion from dry land to paddy field, the relationships between relevant modules with conductivity changed from positive to negative correlation. The relationships between relevant modules with soil EC and AK changed from significantly negative correlation to no correlation. The OM showed insignificantly positive correlations with relevant modules.

**Figure 8:**
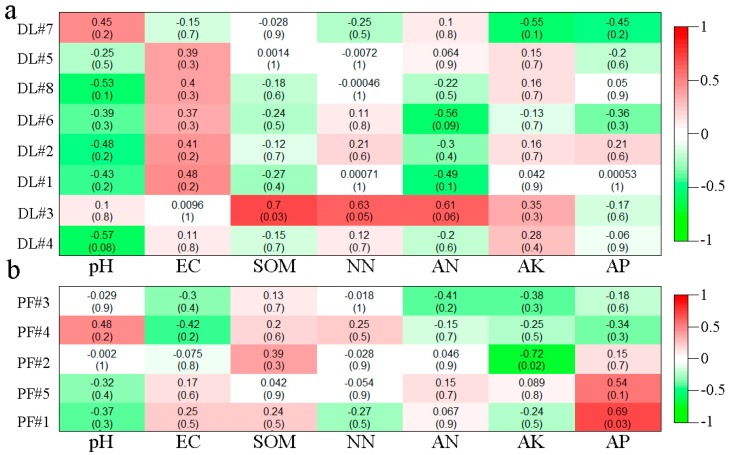
Heatmap analysis of fungal module correlation with environmental factors in DL (**a**) and PF (**b**).

Similarly, the relationships between the modules with more than 5 nodes and the environmental factors in the fungal molecular ecological network were analyzed by heatmap. The as-derived heatmap structure is shown in Figure 8. For the dry land fungal network modules, module 3 had significantly positive correlations with OM, NN and ammonia nitrogen, and module 6 had a significantly negative correlation with ammonia nitrogen (Figure 8a). No significant correlations have been found between other environmental factors and modules. On the other hand, for the paddy field, module 2 had a significantly negative correlation with soil AK, and module 1 had a significantly positive correlation with AP (Figure 8b). No significant correlations have been found between other environmental factors and modules. The significance levels between the modules of paddy field and environmental factors were lower than those between modules of dry land and environmental factors, which may be due to the great changes in each environmental factor and unstable initial relationships between microbial modules and environmental factors in the short time after conversion. The driving relationships between microbial species corresponding to OTU in these potential characteristic modules and environmental factors provide a powerful tool for clarifying the response of microbial community structure to environment changes, and are beneficial for the development of new strains to adapt to the new environment.

#### 3.3.2. Interactions between Soil Microbial Species and Environmental Factor Network

**Figure 9:**
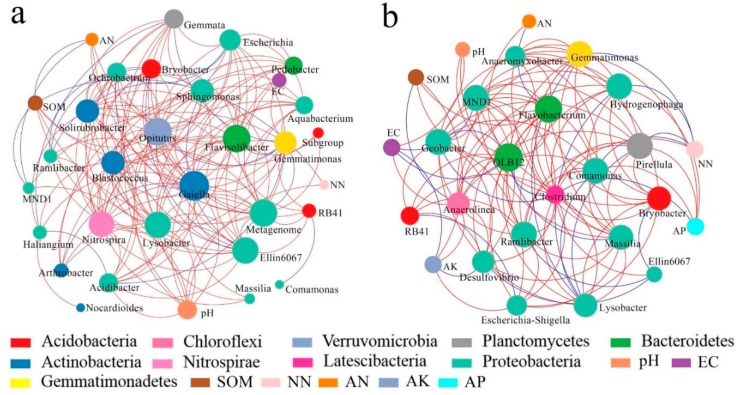
Molecular ecological networks of dominant bacterial species and environmental factors in DL (**a**) and PF (**b**).

Furthermore, the network interactions between soil microbial species and environmental factors, with environmental factors as the nodes, were analyzed. The top 50 genera of soil microorganisms in dry land and paddy field were selected for the construction of an RMT-based network to compare the relationships between environmental factors and microorganisms. The results are shown in Figure 9. *Proteobacteria* was the primary species in the constructed networks of dry land and paddy field. *Actinobacteria* was the dominant species in the constructed network of dry land, but it was not involved in the network of paddy field (Figure 9a). Among the interactions between the dominant bacterial species and environmental factors, the numbers and strengths of nodes connected to soil OM, pH, and EC were the highest. The interactions between AK and AP in paddy field and bacteria were close, showing that soil nutrients such as AK and AP are the main factors promoting the development of bacteria in paddy field (Figure 9b). In contrast, no connections were found between dominant species and environmental factors in the bacterial interaction network of dry land. The connections between soil physical and chemical properties and bacterial species showed that the interactions in this network were dominated by negative correlations. In the interaction network, the environmental factors were located on the perimeter, were closely connected with the dominant species, and affected the critical species located amid the network. In conclusion, OM, pH and EC were more frequently connected in the network of dry land, indicating that OM, pH and EC are the determining factors for the changes of bacterial community structures during the process of dryland-to-paddy conversion.

**Figure 10:**
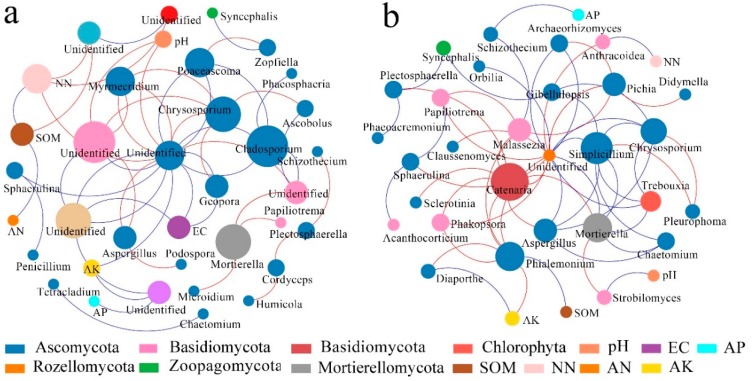
Molecular ecological networks of dominant fungal species and environmental factors in DL (**a**) and PF (**b**).

In relation to the interactions between species and environment, *Ascomycota* was the primary species in the ecological networks of dry land and paddy field (Figure 10). *Basidiomycota* was the dominant species in the interaction fungal network of paddy field, but *Basidiomycota* was not involved in the interaction network of dry land, where the numbers and strengths of nodes connected to NN, OM, and EC were the highest, followed by pH and AK, ammonia nitrogen, and AP, successively (Figure 10a). In the fungal network of paddy field, fungi had a significant correlation with AK, and had positive correlations with OM, pH, ammonia nitrogen and AP, but these positive correlations were relatively loose (Figure 10b). In addition, the remaining physical and chemical properties were indirectly related to the dominant species. According to the connections between soil physicochemical properties and soil microbial species, the interactions in dry land fungal networks were dominated by negative correlations, while the interactions in paddy field fungal networks were dominated by positive correlations, demonstrating that the soil bacteria cooperated with each other in order to adapt to the unfavorable stress caused by flooding.

## 4. Discussion

### 4.1. Changes in Microbial Community Structure After Dryland-to-Paddy Conversion

A small change in the soil environment may cause major changes in the global environment [47]. Land use conversion changes soil water, heat, other ecological conditions, and the living environment for the reproduction of microorganisms [2,23], greatly influencing the carbon and nitrogen cycles in soil. Soil microorganisms are sensitive to changes in land use patterns and soil environment [16,17]. In this study, the conversion from dry land to paddy field showed a great impact on soil microbial diversity and community structure. After the conversion, in a short time, the diversity of soil microorganism was decreased, and the Shannon and Simpson indices in paddy field were significantly lower than those in dry land. Previous studies also reported that soil environment was unstable in a short time after land consolidation [48,49,50,51]. Generally, with the extension of land consolidation period, the microbial diversity increases gradually, but the diversity decreases in the short term after the consolidation [52,53,54,55]. Meanwhile, soil microorganisms have a certain selective adaptability to soil environment. Zhang et al. reported that the moisture content in soil had a significant influence on the quantity, structure and diversity of soil microbial communities [56]. NMDS analysis indicated that the microbial communities in farmland soil were largely changed after the conversion from dry land to paddy field, which might be related to the changes of soil environmental conditions after flooding [57]. More studies have suggested that water stress could lead to changes in the abundance and community structure of soil microorganisms, and the variance in moisture content in soil could lead to changes in pH, EC, OM and other factors in farmland [58,59,60]. These soil properties have been confirmed to be important reasons for changes in soil microbial abundance and community structure [59,60,61,62], as also evidenced by the results of the present study.

The diversity of the soil microbial community was reduced iIn a short time by the conversion, and the relative abundance of bacterial and fungal microbial communities was affected to varying degrees. Forthe bacteria, the dominant bacterial community was *Proteobacteria* [63], with an abundance of greater than 30%, with similar levels being found in most farmland soils. For instance, Jangid reported that *Proteobacteria* was found, with a prevalence of 30.6–43.2% [64], irrespective of farming patterns. In the present study, the abundance of aerobic microorganisms such as *Actinobacteria* decreased significantly, while that of anaerobic microorganisms such as *Chloroflexi* and *Firmicutes* increased significantly. For instance, *Firmicutes* is mainly distributed in anaerobic environments such as biogas fermentation and high-temperature composting, with high environmental adaptability [65], and the abundance of *Firmicutes* increased from 0.23% to 18.85% after the conversion from dry land to paddy field. The conversion led to significant changes in the phylum level of the soil microbial community. Many studies show that soil bacteria are closely associated with their environmental factors [59,60,61,62]. For example, Shen et al. reported that soil pH was the dominant factors affecting microbial community structure and diversity [59]. In the present study, the Mantel test results showed that soil pH, EC and OM were important driving factors determining the changes in characteristics of the bacterial community. The changes in soil environmental conditions caused by the consolidation of farmland led to the changes in composition and diversity of microbial community.

For the fungi, *Ascomycota* was the most dominant fungal species in dry land, accounting for about 45.74%. *Ascomycota* is mostly a saprophytic fungus in Nature. The main function of saprophytic microorganisms is to decompose refractory organic matter and to improve the contents of organic matter and nutrients in the soil. Saprophytic microorganisms play an important role in carbon and nitrogen cycles [66]. *Basidiomycota* mostly grows in rotten wood, compost and other humid environments [66,67,68], and the abundance of *Basidiomycota* was increased from 7.93% in dry land to 22.91% (dominant) in paddy field. Most studies found that fungal community structures were usually closely related to soil nutrient contents [67,68,69]. In the present study, compared to dry land, paddy field was flooded for a long period, and most of the voids in soil were occupied by water, resulting in a low oxygen content and weak redox property of the soil [25,70]. Therefore, the activity of soil was weakened, and with it the ability of microorganisms to capture soil nutrients such as carbon and nitrogen was hindered, affecting the growth and metabolism of microorganisms and causing changes in the structure and function of fungi. Microorganisms are very sensitive to environmental variations [17,64], and the great environmental difference between dry land soil and paddy field would affect the carbon and nitrogen cycle process involving microorganisms.

### 4.2. Changes in the Structure of Microbial Molecular Ecological Networks After Dryland-to-Paddy Conversion

In Nature, species play an important role in mass circulation and energy flow by forming complex networks [37,38]. In this study, molecular ecological networks were innovatively applied to the study of soil microbial community structure and function before and after the conversion from dry land to paddy field. It was found that the conversion changed the interactions between microorganisms in the molecular ecological networks. Generally speaking, the more stable an ecosystem is, the more complex the ecosystem structure will be [36]. The results of the present work agreed with this principle. The dry land had been cultivated for many years continuously, and a stable ecological structure had been formed. Compared to the dry land, in a short time after the conversion, the bacterial and fungal networks in the paddy field had decreased in size, and the relationships between species were simpler, which may be related to the stress after flooding. The topological characteristics of network nodes showed that the interactions between microorganisms in dry land and paddy field were different, and the abundance and diversity of species corresponding to the critical OTU and OTU values in the entire networks were different.

Soil microorganisms are sensitive to land use patterns and environmental changes [20,24]. In this study, we found the conversion of dryland-to-paddy greatly impacted the structure of the bacterial and fungal molecular ecological network. It was found that the soil bacterial molecular ecological network connection in the dry land was more complex, with more nodes and connection lines. Moerover, the connections within and between modules were closer. The *Actinobacteria* played a dominant role in bacterial networks of both dry land and paddy field. Zhou et al. found that, the soil bacterial network structure changed, and *Actinobacteria* played a key role, as the atmospheric CO_2_ concentration increased [40]. In the bacterial network of paddy field, anaerobes such as *Firmicutes* also played an important role. In the process of land consolidation, land use conversion causes differences in management measures and farmland environmental factors [23,71], and thus affects the uniqueness and diversity of bacterial community structure in soil. The interaction between dominant species and the environment demonstrated that, soil OM, pH and EC were dominant, affecting the microbial community. The results implied that waterlogging stress in paddy fields changed the soil pH, water and nutrients in different level, as well as affecting the development of soil bacterial communities. Previous studies proved that the evolution of microbial community structure and function was closely related to the levels of carbon and nitrogen in the soil, and the level of one element might limit the uptake and utilization of other elements by microorganisms [28,63,72]. The dryland-to-paddy conversion could improve the availability of soil nutrients through changing the physical and chemical properties of the soil, and thus changed the network structure of the microorganisms.

In the fungal molecular ecological network, *Ascomycota* play a lead role in the fungal networks of both dry land and paddy field, and the fungal communities presented mainly negatively correlation among themselves (Figure 6), which indicated the underlying competition in resources. The fungal network modular structure of dry land, with a greater number of nodes and connecting lines, was more complex than that of paddy field. The changes in the fungal network module structure may be attributed to the changed soil physical structure due to dryland-to-paddy conversion. Because the porosity of soil would be changed by flooding, redox reactions would be thus affected [24], leading to the changes in microbial community structure. According to the work of Hannula et al., changes in soil environment is an important factor affecting the structure and function of a fungal community [73]. Module 3 in the network of dry land (Figure 8) had significant correlations with OM, AN and NN. The interaction networks of soil fungi and environmental factors in dry land and paddy field were totally distinct, but nutrients including OM, AK and NN were the main factors affecting the development of soil fungi. Many studies reported that the structure of a fungal community was usually closely related to the content of nutrients in the soil [74], and this principle was also found in the present study.

The average connectivity values of bacterial and fungal networks in paddy soil were smaller than those in dry land, indicating that the microorganisms in paddy soil were more sensitive. When the environment is changed, the environmental disturbance will affect the whole ecological network after a period of time [75], and the instability of the network structure increases. Generally, the higher the content of effective nutrients in an ecosystem, the higher the complexity and stability of the microbial ecological network [35,38]. The high stability of communities is an important factor in the functioning of an ecosystem [36]. Although a large area of conversion from dry land to paddy field can ensure the balance between occupation and compensation and maintain food security, the resulting instability of farmland ecosystems should be a serious concern. During the conversion process, the occurrence of these microbial interactions on a microscopic scale still requires further study. In the future, the dynamic variations of microbial networks in the conversion processes from dry land to paddy field should be continually investigated.

## 5. Conclusions

Land use is the most direct activity of human beings to interfere with soil carbon and nitrogen cycles in ecosystems. It is very important to comprehensively and systematically understand the changes in soil microbial communities in the process of land use conversion. In this study, pot-based experiments, high-throughput sequencing and molecular ecological network analysis were used to explore the changes in soil environment and microbial communities during the conversion process from dry land to paddy field:

(1) The abundance and diversity of soil bacteria and fungi decreased in a short time after the conversion process; the most dominant soil bacterial species in dry land and paddy field were *Proteobacteria*, and the most dominant fungal species in dry land and paddy field were *Ascomycota* and *Basidiomycota*, respectively. The abundance values of *Actinobacteria*, *Firmicutes*, and *Olpidiomycota* were greatly changed after the conversion, and these species can serve as indicator species for the evolution of soil microbial community diversity in the conversion process.
(2) The dryland-to-paddy conversion had a great influence on the structure of microbial molecular ecological networks. Compared to dry land, the microbial interaction network modular structure and interspecific relationships of paddy field were simpler. The number of nodes, number of connecting lines, average connectivity and clustering coefficient of the soil microbial network of paddy field were lower. When the environment changed, the disturbance of environmental factors would affect the whole ecological network after a period of time, and the instability of network structure would increase. In the future, the dynamic variations of microbial network in conversion processes from dry land to paddy field should be continually investigated.
(3) The soil microbial community structure was significantly related to environmental factors. The changes in pH, EC, OM and AK caused by the conversion were the main reasons for the change of soil microbial community structure (*p* < 0.05). The soil microbial community structure was closely related to the dominant microorganism species, and the soil environmental factors were the critical factors limiting the adaptive development of microorganisms.

## Figures and Tables

**Table 1:** Comparison of the topological properties of molecular ecological networks in DL and PF.

Parameters	Bacteria		Fungi	
	DL	PF	DL	PF
**Similarity threshold**	0.88	0.88	0.86	0.86
**Nodes**	261	160	126	70
**Links**	637	223	186	83
**Average degree (AvgK)**	4.881	2.788	2.952	2.371
**Average clustering coefficient (AvgCC)**	0.291	0.309	0.145	0.135
**Average path distance (AvgPD)**	6.36	5.699	4.362	4.006
**Density**	0.019	0.018	0.024	0.034
**Connectivity**	0.692	0.275	0.724	0.494
**Number of modules**	29	28	13	14
**Modular index**	0.694	0.822	0.659	0.678
**R^2^**	0.814	0.848	0.856	0.859

**Table 2:** Mantel Test between soil microbial community at the OTU level and environmental factors in DL and PF.

Soil Properties	Bacteria		Fungi	
	DL	PF	DL	PF
pH	0.438 *	0.439	0.262 *	0.189
EC (mS·cm^−3^)	0.350 *	0.597	0.260 *	0.234
OM (g·kg^−1^)	0.287 *	0.330 *	−0.168	−0.098
NN (mg·kg^−1^)	0.254	0.129	−0.059	−0.474
AN (mg·kg^−1^)	0.308	0.097	0.028	0.156
AP (mg·kg^−1^)	0.138	−0.054	−0.117	0.045
AK (mg·kg^−1^)	−0.196	0.157	−0.083	0.397 *

Note: * represents a *t*-test of *p* < 0.05 between different treatments. EC, electrical conductivity; OM, organic matter; NN, nitrate nitrogen; AN, ammonical nitrogen; AP, available phosphorus; AK, available potassium. The same abbreviations are used below.
